# Dipeptidyl peptidase-4 and kidney fibrosis in diabetes

**DOI:** 10.1186/s13069-016-0038-0

**Published:** 2016-02-13

**Authors:** Sen Shi, Daisuke Koya, Keizo Kanasaki

**Affiliations:** Department of Diabetology and Endocrinology, Kanazawa Medical University, Uchinada, Ishikawa 920-0293 Japan; Division of Anticipatory Molecular Food Science and Technology, Kanazawa Medical University, Uchinada, Ishikawa 920-0293 Japan; The Department of Vascular and Thyroid Surgery, The Affiliated Hospital of Luzhou Medical College, Luzhou, 646000 People’s Republic of China

**Keywords:** Diabetic nephropathy, Dipeptidyl peptidase-4, Kidney protection

## Abstract

Diabetic nephropathy (DN) is the most common cause of end-stage kidney disease worldwide and is associated with increased morbidity and mortality in patients with both type 1 and type 2 diabetes. Recent evidence revealed that dipeptidyl peptidase-4 (DPP-4) inhibitors may exhibit a protective effect against DN. In fact, the kidney is the organ where the DPP-4 activity is the highest level per organ weight. A preclinical analysis revealed that DPP-4 inhibitors also ameliorated kidney fibrosis. In this review, we analyzed recent reports in this field and explore the renoprotective effects and possible mechanism of the DPP-4 inhibitors.

## Background

Diabetes mellitus has become a major global health issue [1].The number of people with diabetes worldwide is expected to rise from 382 million in 2013 to 592 million by 2035, according to the International Diabetes Federation [2]. Diabetic nephropathy (DN) is one of the most devastating complications of diabetes [3, 4]. The risk of DN is tightly linked to poor glucose control in both type 1 and type 2 diabetes, which is associated with hyperglycemia [5, 6], and the impacts of hyperglycemia are generally mediated through diverse metabolic pathways, including increased reactive oxygen species formation, excessive production of advanced glycation end products (AGEs), and the activation of the polyol, protein kinase C (PKC), and hexosamine pathways [7]. The activation of these pathways leads to a complex dysregulation of various effector molecules, resulting in cellular damage and dysfunction [7]. Experimental studies have shown that some of these pathophysiological mechanisms may be modified by dipeptidyl peptidase-4 (DPP-4) inhibition [8, 9], and preclinical studies also suggest that DPP-4 inhibitors provide renoprotection above and beyond lowering the glucose levels through its protein-protein interactions and proteolytic and antioxidant properties [10]. In this review, we focus on the possible mechanisms by which DPP-4 inhibitors combat diabetic nephropathy, especially about kidney fibrosis.

## Biology of DPP-4

DPP-4 is a cell surface aminopeptidase that was originally characterized as a T cell differentiation antigen (CD26). It is a multifunctional protein that exerts diverse biological activities, such as protease activity, association with adenosine deaminase (ADA), interaction with the extracellular matrix, cell surface co-receptor activity to mediate viral entry, and regulation of intracellular signal transduction coupled to the control of cell migration and proliferation [11–15]. DPP-4 is expressed ubiquitously and found in many cell types, including the endothelial cells in multiple vascular beds, rendering the enzyme highly accessible to the peptide substrates circulating through the gut, liver, lung, and kidney [16].

DPP-4 is a member of the serine peptidase/prolyl oligopeptidase gene family. The members of this family are often classified into subgroups according to their structure and function and include: the membrane-bound peptidases: fibroblast activation protein (FAP)/seprase; the resident cytoplasmic enzymes: DPP-8 and DPP-9; and the nonenzymatic peptidases: DPP-6 and DPP-10 [17]. The complexity of DPP-4’s action is amplified by the panoply of bioactive DPP-4 substrates, which, in turn, act as elegant biochemical messengers in multiple tissues, including the immune and neuroendocrine systems. More than 30 peptide substrates for DPP-4 have been identified, including glucagon-like peptide-1(GLP-1), glucose-dependent insulinotropic peptide (GIP) [17], brain natriuretic peptide 1–32 [18, 19], neuropeptide Y [20], high mobility group protein 1 (HMGB1) [21], and others [20, 22, 23].

**Fig. 1:**
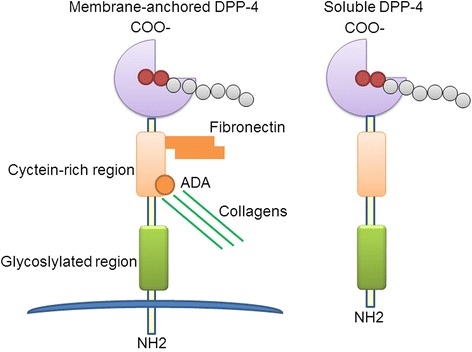
Membrane-anchored DPP-4 and soluble DPP-4. Catalytically active DPP-4 is liberated from the plasma membrane to produce a soluble circulating form, sDPP-4, which lacks the intracellular tail and transmembrane regions and accounts for a substantial proportion of DPP-4 activity. In addition to its exopeptidase activity, DPP-4 also functions as a binding protein which can bind with fibronectin and adenosine deaminase (ADA)

DPP-4 transmits signals across the cell membranes and interacts with other membrane proteins. The DPP-4 gene encodes a type II transmembrane protein of 766 amino acids, which is anchored to the lipid bilayer by a single hydrophobic segment located at the N-terminus and has a short cytoplasmic tail of six amino acids [24] (29). The extracellular portion of DPP-4 contains a glycosylation domain, a cysteine-rich domain, and a catalytic domain [17]. Mutation studies demonstrated that the C-terminal loop of DPP-4 is essential for both dimer formation and catalytic efficacy [25]. An analysis of the crystal structure revealed that DPP-4 can also form tetramers between two soluble DPP-4 proteins and two membrane-bound DPP-4 proteins. These interactions may influence the efficiency of the entry and cleavage of substrates by the catalytic site or allow cell-cell communication [25]. Catalytically active DPP-4 is liberated from the plasma membrane to produce a soluble circulating form, sDPP-4 (727 aa), which lacks the intracellular tail and transmembrane regions and accounts for a substantial proportion of DPP-4 activity in human serum [26, 27]. Membrane-bound DPP-4 contains residues 1–766, whereas sDPP-4 contains residues 39–766. sDPP-4 lacks the cytoplasmic domain [residues 1–6], transmembrane domain [residues 7–28], and the flexible stalk [residues 29–39] [17, 26] (Fig. 1). sDPP-4 can activate intracellular signaling pathways and increases the proliferation of human lymphocytes, independent of either its catalytic activity [28] or binding to ADA [28]. sDPP-4 impairs insulin-mediated activation of Akt in the human adipocyte, skeletal muscle, and smooth muscle cells in vitro [29]. However, the mechanisms by which sDPP-4 activates signal transduction are poorly understood.

DPP-4 activity is subject to regulation at many levels, including control of gene and protein expression, interactions with its binding partners, and modulation of its enzyme activity. The importance of cytokines in regulating DPP-4 activity was demonstrated in chronic B lymphocytic leukemia cells, leading to the upregulation of both intracellular and cell surface DPP-4 expression, as well as DPP-4 activity [30]. Cell surface and intracellular DPP-4 expression is also highly regulated; it is often expressed at low levels under basal conditions, and then markedly induced by stimulation, such as T cell activation by mitogenic or antigenic stimuli [31]. The control of DPP-4 shedding, which generates sDPP-4, is poorly understood. Lamers et al. found that TNF-α and insulin increased the release of sDPP-4, although there were no changes in the expression of the DPP-4 mRNA in human adipocytes isolated from visceral depots [29]. Some other studies have focused on the origin of sDPP-4 and found that kidney extracts exhibited the highest DPP-4 activity; however, the most important source of sDPP-4 is the bone marrow and not the kidney [32]. These studies highlight our limited understanding of the cell types and tissues that contribute to the generation of sDPP-4 activity in the plasma in vivo. The characterization of these cells, evaluation of additional sources for sDPP-4, and the mechanism of release require additional studies.

## The role of DPP-4 inhibitors in diabetes and kidney protection

In general, incretins are a group of metabolic hormones that stimulate a decrease in the blood glucose levels, by either increasing insulin release or reducing gastrointestinal absorption. The prototypical incretins are the intestinal GLP-1 and GIP hormones, and both GLP-1 and GIP are rapidly inactivated by the enzyme DPP-4. Therefore, DPP-4 is a well-documented drug target for the treatment of type 2 diabetes [33–35]. The pharmacological inhibition of DPP-4 results in GLP-1 accumulation, which stimulates insulin secretion and contributes to the reduction of postprandial hyperglycemia. Recently, the beneficial pleiotropic effects of DPP-4 inhibitors were reported in both clinical research and preclinical experiments.

Two types of DPP-4 inhibitors have been used in the clinic: dipeptide structure mimetics and non-peptidomimetics. The first type includes sitagliptin (approved by FDA in 2006), vildagliptin (approved by European Medicines Agency in 2007), and saxagliptin (approved by FDA in 2009), while the non-peptidomimetics include linagliptin (xanthine-based, approved by FDA in 2011) and alogliptin (modified pyrimidinedione, approved by FDA in 2013). These are all small molecules that are rapidly absorbed following oral dosing, resulting in greater than 80% inhibition of the DPP-4 activity over a full 24-h period. Typically, these drugs raise the peripheral plasma concentration of the intact forms of both incretins by two- to three-fold. Although this class differs widely in their chemistry, they are all selective for DPP-4 [22]. Clinical proof of concept for DPP-4 inhibition in diabetic patients was first reported in 2002, where a DPP-4 inhibitor significantly reduced the fasting plasma glucose and HbA1c levels in a 4-week study [36]. DPP-4 expression positively correlates with the amount of visceral adipose tissue, adipocyte size, inflammation, and HbA1c levels, and negatively correlates with the glucose infusion rates during euglycemic-hyperinsulinemic clamp [37, 38]. The efficacy of the DPP-4 inhibitors in reducing glycemia is weaker than that of sulfonylureas, insulin, and thiazolidinediones, but they are significantly better tolerated and do not produce weight gain [39–41]. Furthermore, DPP-4 inhibitors not only have benefits for patients who have recently developed diabetes [42] but also in patients with long-standing diabetes [43].

In addition to their glucose-lowering action, DPP-4 inhibitors have been demonstrated to play a protective role in cardiovascular diseases, including hypertension [44], abdominal aortic aneurysm [45], cardiomyopathy [46], atherosclerosis [47], and peripheral vascular disease [48], via both GLP-1-dependent and GLP-1-independent pathways due to their diverse, widely distributed, and pleiotropic actions [49]. Many in vivo and in vitro studies also found that DPP-4 inhibitors can prevent organ fibrosis, including cardiac fibrosis [50, 51], hepatic fibrosis [52], and kidney fibrosis [53]. DPP-4 is localized on the surface of many cell types, including the endothelial cells, kidney epithelial cells, and T cells, where they have a binding partner and transmit intracellular signals [54]. In fact, the kidney expresses the highest levels of DPP-4 per organ weight [20]. Moreover, the mammalian kidney has high concentrations of DPP-4 [20], and the expression of DPP-4 is increased in cultured human renal glomerular epithelial cells during inflammation [55] and in a rat model of type 2 diabetes mellitus [56]. Several candidates for the GLP-1-independent effects of DPP-4 inhibitors in the kidney have been identified and include the known substrates of DPP-4 cleavage, such as HMGB1, Meprin β, neuropeptide Y (NPY), and peptide YY (PYY) [57]. Thus, some researchers have demonstrated that increased DPP-4 activity in the kidney or urine is a hallmark for human glomerular diseases [54, 58]. Some of the DPP-4 inhibitors were analyzed to confirm their role in the kidney. In animal models, a reduction of albuminuria and an improvement in the histological changes in the kidney were observed in T1DM models treated with vildagliptin [59] and in T2DM models treated with sitagliptin [60]. A significant reduction in urinary albumin excretion was also observed in diabetic endothelial nitric oxide synthase knockout mice treated with linagliptin in addition to an Ang II receptor antagonist [8]. In the experimental model of renal ischemia/reperfusion injury treated with vildagliptin, DPP-4 inhibition produced nephroprotective effects that were mediated by antiapoptotic, anti-inflammatory, and anti-oxidative effects [61]. Linagliptin is the recently approved DPP-4 inhibitor and exhibits non-linear pharmacokinetic properties, with a less than dose proportional profile and is almost completely eliminated via the enteric system, with less than 5% found in urine [62]. In humans, it was shown that linagliptin significantly reduced the urinary albumin excretion of patients with T2DM after 24 weeks of treatment [63]. The pharmacokinetic profiles after linagliptin administration showed the accumulation t1/2 of linagliptin following multiple 5 mg/day doses, and there were no significant changes in the control and diabetic subjects, regardless of their renal function [64]. The antifibrotic properties of DPP-4 inhibitors have also been shown in other models of kidney fibrosis, such as the unilateral ureteral obstruction (UUO) model, where the administration of a novel DPP-4 inhibitor, LC15-0444, resulted in a significant decrease in albuminuria, the urinary excretion of 8-isoprostane, and renal fibrosis [65]. In our newest study, we found that linagliptin restored the normal kidney structure of streptozotocin (STZ)-induced diabetic kidney fibrosis in CD-1 mice without altering the blood pressure, body weights, blood sugar levels, or organ weights (of the kidney, liver, and heart) compared with the untreated diabetic mice [53].

Transforming growth factor β (TGFβ) is the primary cytokine that drives fibrosis in the kidney and other organs that are susceptible to fibrotic injury, such as the lung and liver. Members of the TGFβ superfamily transduce intracellular signals through Smad proteins. A study demonstrates that TGFβ signals can mediate renal fibrosis through Smad2/3 [66]. DPP-4 inhibitors may ameliorate diabetic nephropathy and reduce the overproduction of TGF-β1. Renoprotection is attributed to the inhibition of DPP-4 activity, which mimics incretin action, and the activation of the GLP-1R [59]. In our previous study, STZ-induced diabetic mice treated with the DPP-4 inhibitor linagliptin exhibited a suppression of DPP-4 activity/protein expression and an amelioration of kidney fibrosis associated with the inhibition of the endothelial-to-mesenchymal transition (EndMT) and TGF-β2-induced Smad3 phosphorylation [53]. EndMT was first discovered in heart development [9], which was confirmed to be crucially important in forming the valves and septa of the heart during embryogenesis [67, 68]. EndMT contributes to the accumulation of activated fibroblasts and myofibroblasts in kidney fibrosis [69], fibroblasts are key mediators of fibrosis in the kidney and other organs [70]. Chronic kidney disease (CKD) is associated with an increase in circulating angiogenesis and NO inhibitors, which impact proliferation and apoptosis of cardiac endothelial cells and promote EndMT, leading to cardiac fibrosis and capillary rarefaction [71]. EndMT is not only involved in kidney fibrosis progress, Zeisberg et al. confirmed that EndMT also involved in heart and tumor progression [72–77]. In this regard, linagliptin could exhibit an antifibrotic effect through a mechanism that specifically targets endothelial cells [53, 78, 79]. DPP-4 is essential for TGF-β-induced receptor hetero-dimerization and subsequent intracellular signal transduction, including the levels of TGF-βRs and the protein-protein interactions of TGF-βRs, both of which are critical for TGF-β signal transduction. We found that the TGF-β2-induced formation of the TGF-βR1/2 heterodimer was suppressed in the DPP-4 siRNA-transfected endothelial cells compared with the cells transfected with a control siRNA [79]. In a UUO model, a DPP-4 inhibitor, LC15-0444, reduced the levels of inflammatory and fibrotic markers, such as phosphorylated Smad2/3, TGF-β1, toll-like receptor 4, HMGB1, NADPH oxidase4, and nuclear factor kappa B [65]. These results suggest that the activation of DPP-4 in the kidney has an important role in TGF-β signaling and the progression of renal disease and that targeted therapy that inhibits DPP-4 may prove to be a useful new approach in the management of progressive renal disease, including kidney fibrosis.

### Interaction of DPP-4 and integrin β1 in kidney fibrosis

Integrins exist as αβ heterodimers that are formed from 18 α- and 8 β-subunits, each of which exhibits different ligand binding and signaling properties [80]. Each integrin subunit consists of an extracellular domain, which determines the ligand binding properties, a transmembrane domain, and a short cytoplasmic tail that binds to multiple cytosolic and transmembrane proteins to form focal adhesions (with the exception of β4) [81]. Integrins bind to extracellular matrix (ECM) glycoproteins, including collagens, fibronectins, and laminins, and cellular receptors, such as vascular cell adhesion molecule-1 (VCAM-1) and members of the intercellular cell adhesion molecule (ICAM) family [82, 83]. In addition, integrins also play key roles in the assembly of the actin cytoskeleton as well as in modulating the signal transduction pathways that control biological and cellular functions, including cell adhesion, migration, proliferation, cell differentiation, and apoptosis [84]. Integrins are able to transduce signals intracellularly following ligand binding (“outside-in” signaling) [85]. Unlike most other cell receptors, integrins can shift between high- and low-affinity conformations for ligand binding (“inside-out” signaling) [86]. Depending on the cell type, integrins can be either basally activated, as is the case in most adherent cells that are attached to a basement membrane, or basally inactive, as is the case with platelets or leukocytes that freely circulate until they are activated to undergo platelet aggregation or mediate an inflammatory response, respectively. Integrins themselves have no kinase activity, but instead provide a connection between the ECM and the actin cytoskeleton. This connection allows integrins to regulate the cytoskeletal organization and cell motility, as well as to alter the fluxes of many intracellular-signaling pathways, including cell survival, cell proliferation, cell shape, and angiogenesis [86, 87]. Thus, integrins are critical for maintaining cellular homeostasis, triggering a number of signaling pathways under normal conditions; under pathological conditions, integrins are associated with a wide variety of renal pathologies, including obstructive nephropathy and DN [88–91].

**Fig. 2:**
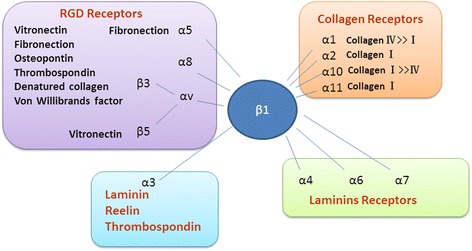
Integrin β1 receptors and their ligands. In the integrin family, the β1-integrin is the most critical, given that β1-integrin can pair with different α-subunits, making it a receptor for many types of stimuli

Among the members of the integrin family, the β1-integrin is the most critical, given that β1-integrin can pair with different α-subunits, making it a receptor for many types of stimuli [92–95] (Fig. 2). Integrin β1 is ubiquitously expressed and can bind to multiple partners, and thus it is not surprising that knockout of β1-integrin results in embryonic lethality due to a complete inhibition of preimplantation development. In contrast, knockouts of the α1, α2, α10, and α11 integrin subunits, which each exclusively heterodimerize with β1 to function as primary collagen receptors, are all viable and fertile, but possess distinct characteristic abnormalities [96]. Focal adhesion kinase (FAK) is the most essential intracellular integrator in the integrin β1-FAK signaling pathway, and the abnormal redistribution and decreased expression of integrin β1 and FAK are important molecular events that regulate the functions of podocytes under abnormal hemodynamic conditions [97]. Expression of integrin β1 by fibroblasts is required for fibrogenesis. Blocking integrin β1 signaling can diminish the progression of cutaneous fibrosis [98]. Yeh et al. found that the expression of the β1-integrin mRNA and protein was significantly upregulated in UUO mice, which was accompanied by a corresponding elevation in the tubular expression of TGF-β1 [90]. The inhibition of β1-integrin signals reduced the TGF-β1 levels and ameliorated fibrosis, demonstrating strong correlations between the expression of β1-integrin within the tubulointerstitium and the presence of tubulointerstitial fibrosis [90]. Hamzeh et al. also found that in the absence of β1-integrin, human proximal tubular cells fail to activate the signaling cascade that would lead to the synthesis of profibrotic proteins and, ultimately, to the development of renal fibrosis. They also showed that cyclic stretch-induced TGF-β1 and fibronectin expression is mediated by β1-integrin through c-Src- and STAT3-dependent pathways in renal epithelial cells [91].

**Fig. 3:**
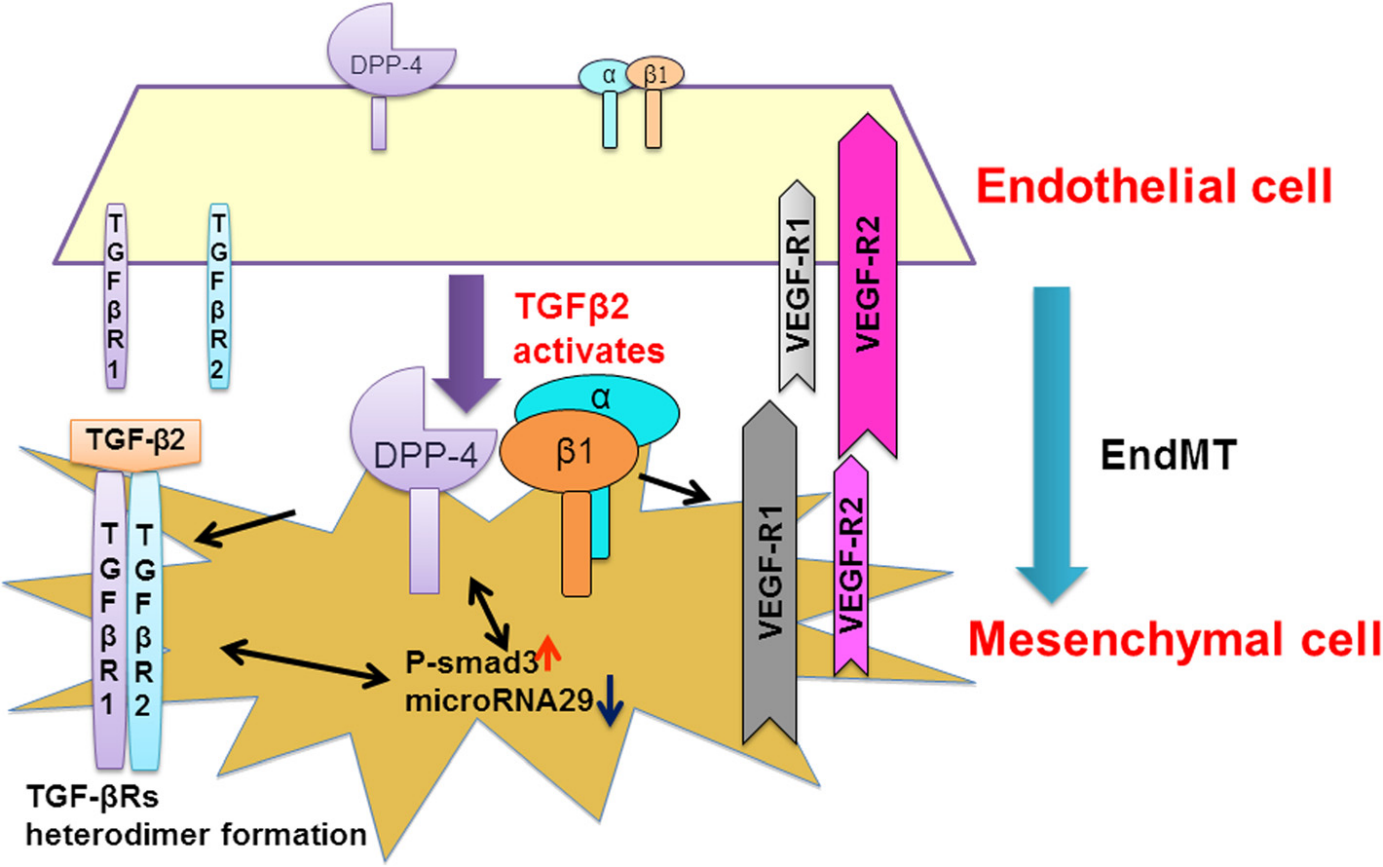
Interaction of DPP-4 and integrin β1 in the endothelial cells. Interaction between DPP-4 and integrin β1 displays key role in the TGF-β-induced signal transductions and VEGF-induced survival signaling in endothelial cells as well as in the subsequent induction of EndMT, which is associated with regulation of miR-29s

The loss of DPP-4 cell surface expression has shown to be associated with decreased phosphorylation of integrin β1 at the S785 residue, which has a key role in cellular adhesion to ECM [99]. In our most recent published paper, we identified a new profibrotic molecular mechanism that was associated with the interaction between DPP-4 and integrin β1 [79] (Fig. 3). The DPP-4-associated EndMT was inhibited by integrin β1 deletion. In addition, DPP-4 or integrin β1 deficiency resulted in the inhibition of TGF-β2-stimulated hetero-dimerization of TGF-βRs. Finally, the interaction between DPP-4 and integrin β1 induced vascular endothelial growth factor-receptor (VEGF-R)1 expression, with the concomitant suppression of VEGF-R2 levels [79]. This is relevant because VEGF is the most prominent stimulus for endothelial cells and angiogenesis. The endothelial cell responses toward VEGF are modulated by distinct VEGF receptors, as VEGFR1 favors the EndMT, whereas VEGFR2 counteracts the EndMT [100]. These results indicate that the interaction between DPP-4 and integrin β1 may be a therapeutic target for kidney fibrosis in diabetes [79].

### MicroRNAs and DPP-4 in the kidney

**Fig. 4:**
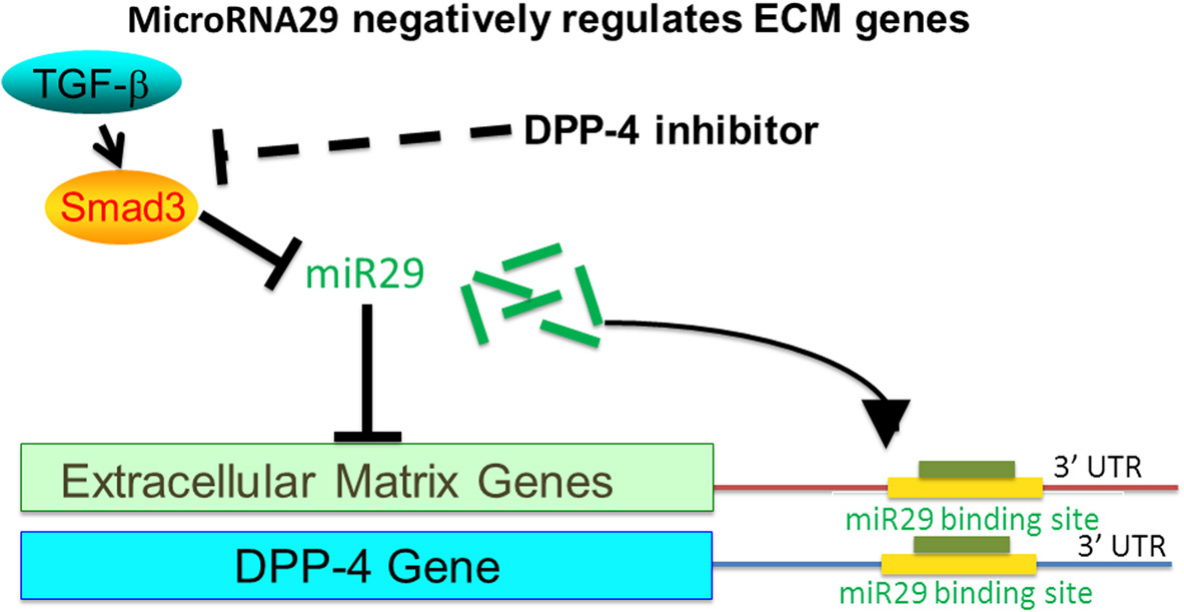
DPP-4 3′UTR and microRNA 29. TGF-β2-stimulated luciferase activity of 3′UTR fragment of DPP-4, where microRNA 29 binding site was involved, DPP-4 inhibitor may restore the miR29 levels by inhibiting TGF-β/Smad3 signaling

**Fig. 5:**
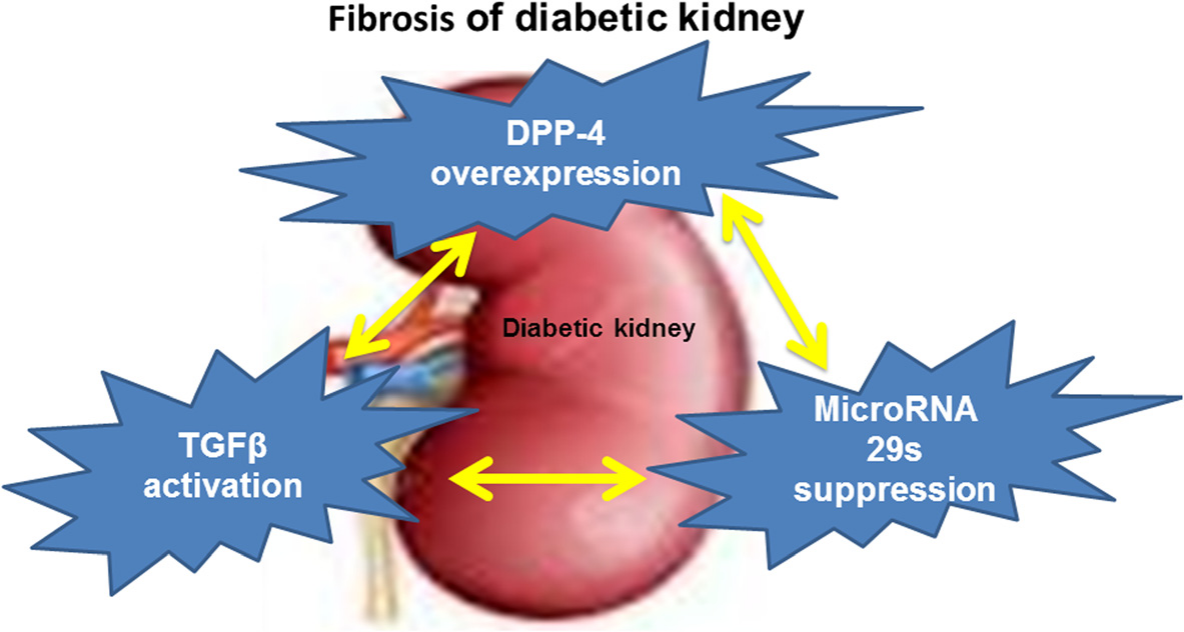
Fibrosis of diabetic kidney. Diabetic kidney fibrosis is associated with suppression of microRNA29s, which targets both DPP-4 protein levels and TGFβ-activating process

The cumulative effects of hyperglycemia, inflammatory cytokines, proteinuria, ageing, high blood pressure, and hypoxia result in alterations of the miRNA expression profiles. The altered miRNA levels initiate a transition program in the normal kidney that ultimately leads to fibrosis. MicroRNAs (miRs) were discovered 20 years ago. The actions and synthesis of miRs are tightly regulated. The key antifibrotic miRs miR-let-7s and miR-29s are involved in suppression and are important for understanding the fibrotic mechanism in the diabetic kidney [53, 101, 102]. According to the prediction of microRNA targets by TargetScan (http://www.targetscan.org/vert_60/), we identified the miR29 bind site in 3′UTR of DPP-4 [53]. By cloning and utilizing the reporter vector containing 3′UTR legends of human DPP-4 mRNA, we have confirmed that miR29 binding site in DPP-4 3′UTR negatively regulated DPP-4 gene expression. In diabetic kidney, the increased DPP-4 levels were associated with the suppression of miR29s when compared with the normoglycemic kidney [53] (Fig. 4). Linagliptin, a DPP-4 inhibitor, ameliorates the kidney functions by inducing miR-29 expression in the diabetic kidney model [53]. A quantitative analysis revealed that microRNAs 29 a, b, and c were suppressed in the diabetic kidney compared with the control kidneys, and linagliptin restored the diabetes-suppressed microRNA 29s levels. Similarly, the TGF-β2-suppressed microRNA29s levels were restored by linagliptin in vitro (Fig. 5). These molecules exhibited similar antifibrotic mechanisms, such as anti-EndMT and anti-TGF-β/Smad signaling effects [53]. A microRNA array analysis of the kidney samples revealed that the expression of the mmu-let-7 family of microRNAs was suppressed in the diabetic kidney [101]. Blockade of FGF signaling induced an EndMT program that can be mimicked by let-7b or let-7c miRNA inhibition [103, 104], and the FGF receptor-microRNA let-7 family axis can suppress the TGF-β receptor I levels [101]. DPP-4 inhibitors have been shown to inhibit the EndMT, and thus may regulate expression levels of miR-let-7s in the diabetic kidney. Additionally, microRNA23 and microRNA 21 have been shown to have an important role in the EndMT and kidney fibrosis [105, 106]; the regulation of these microRNAs by DPP-4 inhibitors must be analyzed to determine the detailed mechanism of kidney fibrosis.

### Perspective

In a Zucker Diabetic Fatty rat model, Takai et al. found there was no significant difference in the blood glucose and plasma insulin concentrations between the sitagliptin- and linagliptin-treated groups, but the DPP-4 activity in the plasma and vascular tissues of the linagliptin-treated group was significantly lower than those in the sitagliptin-treated group [108]. Another study found that CD26/DPP-4 was localized to the nucleus, and its nuclear translocation was enhanced by an anti-CD26 monoclonal antibody, suggesting that DPP-4 inhibition helps the DPP-4 on the cell surface move into the nucleus [109]. These data suggest that DPP-4 can be expressed on the membrane and in the nucleus. Although every DPP-4 inhibitor displays similar role in suppressing DPP-4 activity in the plasma and other tissues, each DPP-4 inhibitor might exert unique, drug-specific effects. Indeed, we have recently reported that Linagliptin can suppress all of the following: DPP-4 activity and protein level, integrin β1 protein levels, EndMT, DPP-4 3’UTR activity, and VEGF-R1 induction/-R2 suppression; Sitagliptin, inhibited none of these [110]. Future studies need to focus on the molecular mechanisms of the DPP-4 inhibitors in different organs and cells.

## Conclusions

The present review describes various aspects and possible mechanisms by which DPP-4 inhibitors combat kidney fibrosis. The activation of DPP-4 in the kidney has an important role in TGF-β signaling, and the progression of renal disease by regulating the microRNA29s levels, and that targeted the inhibition of DPP-4 may prove to be a useful new approach in the management of progressive renal disease, including kidney fibrosis.

## Abbreviations

ADA: adenosine deaminase
AGEs: advanced glycation end products
DN: diabetic nephropathy
DPP-4: dipeptidyl peptidase-4
ECM: extracellular matrix
EndMT: endothelial-to-mesenchymal transition
FAK: focal adhesion kinase
FAP: fibroblast activation protein
GIP: glucose-dependent insulinotropic peptide
GLP-1: glucagon-like peptide-1
HMGB1: high-mobility group protein 1
ICAM: intercellular cell adhesion molecule
NPY: neuropeptide Y
PKC: protein kinase C
TGF-β: transforming growth factor-β
UUO: unilateral ureteral obstruction
VEGF-R: vascular endothelial growth factor-receptor
